# Autophagy controls resource allocation and protein storage accumulation in Arabidopsis seeds

**DOI:** 10.1093/jxb/ery012

**Published:** 2018-02-23

**Authors:** Julien Di Berardino, Anne Marmagne, Adeline Berger, Kohki Yoshimoto, Gwendal Cueff, Fabien Chardon, Céline Masclaux-Daubresse, Michèle Reisdorf-Cren

**Affiliations:** 1 Institut Jean-Pierre Bourgin, INRA, AgroParisTech, CNRS, Université Paris-Saclay, Versailles, France; 2 Université de Versailles Saint Quentin en Yvelines, Université Paris Saclay, Versailles, France; 3 Université Paris-Sud, Université Paris-Saclay, Orsay, France; 10 Swedish University of Agricultural Sciences, Sweden

**Keywords:** 12S globulin, 2S albumin, *atg5*, grain protein content, seed abortion, seed filling

## Abstract

Autophagy is essential for nutrient recycling and plays a fundamental role in seed production and grain filling in plants. Autophagy participates in nitrogen remobilization at the whole-plant level, and the seeds of autophagy mutants present abnormal C and N contents relative to wild-type (WT) plants. It is well known that autophagy (*ATG*) genes are induced in leaves during senescence; however, expression of such genes in seeds has not yet been reported. In this study we show that most of the *ATG* genes are induced during seed maturation in Arabidopsis siliques. Promoter–*ATG8f::UIDA* and promoter–*ATG8f::GFP* fusions showed the strong expression of *ATG8f* in the phloem companion cells of pericarps and the funiculus, and in the embryo. Expression was especially strong at the late stages of development. The presence of many GFP-ATG8 pre-autophagosomal structures and autophagosomes confirmed the presence of autophagic activity in WT seed embryos. Seeds of *atg5* and WT plants grown under low- or high-nitrate conditions were analysed. Nitrate-independent phenotypes were found with higher seed abortion in *atg5* and early browing, higher total protein concentrations in the viable seeds of this mutant as compared to the WT. The higher total protein accumulation in *atg5* viable seeds was significant from early developmental stages onwards. In addition, relatively low and early accumulation of 12S globulins were found in *atg5* seeds. These features led us to the conclusion that *atg5* seed development is accelerated and that the protein storage deposition pathway is somehow abnormal or incomplete.

## Teaser

Autophagy takes place in seeds, as evidenced by detection of autophagosomes and high expression of *ATG* genes in Arabidopsis, and the seeds of the *atg5* mutant exhibit early browning as well as low and early accumulation of 12S globulin.

## Introduction

Macroautophagy (hereafter termed autophagy) is a universal degradation mechanism that facilitates the clearing of unwanted constituents from eukaryotic cells. Autophagy comprises the formation of a cytosolic double-membrane vesicle, termed an autophagosome, that engulfs and sequesters cytoplasmic material such as damaged organelles and protein aggregates prior to their degradation (Liu and Bassham, 2012). Autophagy is essential for the recycling of cellular material and controls nitrogen remobilization at the whole-plant level. In Arabidopsis and maize, autophagy is essential for grain filling (Guiboileau *et al.*, 2012; Li *et al.*, 2015; Masclaux-Daubresse *et al.*, 2017). The autophagy machinery needs the products of many *AUTOPHAGY* (*ATG*) genes. *ATG* genes were discovered by the pioneering work of Professor Yoshinori Ohsumi (Nobel Prize in Physiology or Medicine, 2016), which consisted of screening autophagy-defective yeast mutants under starvation conditions (Tsukada and Ohsumi, 1993). Yeast homologous genes have been found in plants and animals for almost all the *ATG* genes. Among the 50 *ATG* genes discovered in yeast, 18 are part of the central autophagy machinery (Yoshimoto, 2012; Yang and Bassham, 2015) and are absolutely essential for the formation of autophagosomes. In Arabidopsis, about 40 *ATG* genes, among which most are in the core machinery list, have been identified. They are either single genes or members of family genes (Zientara-Rytter and Sirko, 2016), but not all of them have been functionally characterized (Yoshimoto, 2012). *ATG5* gene is a unique copy gene in all animal and plant species studied so far. It is involved in the ATG5–ATG12 conjugation system that is essential for the formation of the ATG8–PE (phosphatidylethanolamine) conjugate that characterizes the autophagosome membrane (Yoshimoto *et al.*, 2004). Arabidopsis *atg5* mutants have been characterized by many research groups (Thompson *et al.*, 2005; Yoshimoto *et al.*, 2009; Guiboileau *et al.*, 2012; Lee *et al.*, 2013; Minina *et al.*, 2013; Izumi *et al.*, 2014; Sakuraba *et al.*, 2014) and they present the typical phenotype of many other *atg* mutants (Doelling *et al.*, 2002; Phillips *et al.*, 2008; Yoshimoto *et al.*, 2009; Wang *et al.*, 2011; Michaeli *et al.*, 2016), namely smaller rosette size, hypersensitivity to N and C starvation, reduced yield, and defects in nitrogen remobilization to the seeds. Using ^15^N tracer experiments, Guiboileau *et al.* (2012) showed that *atg5*, as well as *atg9* and *atg18a*, were strongly affected in N remobilization to the seeds. Despite its lower N remobilization from source leaves, seeds of *atg5* (and of other *atg* mutants) presented higher percentage N content than those of the wild-type (WT), suggesting that in *atg5* the seed sink strength for N relied on N sources originating from post-flowering uptake (Guiboileau *et al.*, 2012). Differences in seed quality between *atg* and WT could be due to the strong effect of the *atg* mutations on the leaf metabolism of C and N, including photosynthesis and N remobilization (Guiboileau *et al.*, 2013; Masclaux-Daubresse *et al.*, 2014). However, it could also be due to seed metabolism and development. Indeed, transcriptome data that can be consulted on public databases such as the BAR (http://bar.utoronto.ca) or Genevestigator (https://genevestigator.com/gv/) sites shows that most of the Arabidopsis *ATG* genes are up-regulated in rosettes with ageing and in seeds during development. This led us to hypothesize that autophagy could play a physiological role in the seed, independently of its role in the mother plant.

In this study, seed development and seed protein contents were monitored in order to identify differences in N resource management in *atg5* by comparison with WT seeds. In order to disconnect the effects of the senescence and autophagy processes, the *sid2-atg5* double-mutant was used in this study. This mutant is affected in salicylic acid synthesis and exhibits a delayed senescence and a higher seed biomass compared to the single *atg5* mutant, but an earlier senescence and a lower seed biomass compared to the Col wild-type (Guiboileau *et al.*, 2012). Because seed filling and development can be influenced by the metabolism of the mother plant, and since autophagy is highly sensitive to nutrient availability, the seed phenotypes were monitored on seeds obtained from plants grown with ample (high) or limiting (low) nitrate supplies. Comparison of the phenotypes obtained in these conditions facilitates the identification of features related to mother-plant resource supply.

## Material and methods

### Plant material and culture conditions

Seeds of *Arabidopsis thaliana* (L.) Columbia wild-type (WT) and the *atg5* (SALK_020601) mutant were obtained from Yoshimoto *et al.* (2009). Transformed seeds expressing the promoter Prom–*ATG8*::*ATG8*::*UIDA* constructs published by Sláviková *et al.* (2005) were kindly provided by Prof. Gad Galili (Weizmann Institute of Science, Rehovot, Israel).

Dry seeds were stratified for 48 h at 4 °C in the dark and then sown on a sand substrate according to Masclaux-Daubresse and Chardon (2011). Plants were cultivated in a growth chamber with short-day conditions (8 h light, 16 h dark) for 55 d after sowing in order to promote rosette development. Then, plants were transferred to long-day conditions (16 h light, 8 h dark) to induce flowering. Hygrometry was maintained at 65%. Plants were cultivated under low nitrogen nutrition (2 mM nitrate) or under high nitrogen nutrition (10 mM nitrate) as described in Masclaux-Daubresse and Chardon (2011).

Pools of 100 seeds were dried at 70 °C for 24 h and weighed on a microbalance (XS3DU, Mettler Toledo, Viroflay, France).

### Cloning and plant transformation

The Prom–*ATG8f*::*GFP* (green fluorescent protein) construct was created using the Gateway® technology (ThermoFisher Scientific). The *ATG8f* (At4g16520) promoter (1827 kb) was amplified by high-fidelity Taq (Phusion High-Fidelity DNA Polymerase, ThermoFisher Scientific) by PCR, using the primers listed in Supplementary Table S1 at *JXB* online, and flanked with attL recombination sites. pDONR207 (Invitrogen) was used as the entry vector and pGWB4 (Nakagawa *et al.*, 2007) was used as the expression vector for the agro-transformation.

The Prom–*Actin*::*GFP::ATG8f* construct was created using a pEZS-CL vector (S. Cutler and D. Ehrhardt, Carnegie Institution for Science, Stanford, CA) whose 35S promoter region was replaced by the *ACTIN2* (At3g18780) promoter. A digestion with the restriction enzymes *Sac*I and *Nco*I was performed to remove the 35S promoter from the plasmid. The sequence of the *ACTIN* promoter was amplified with primers that provided compatible ends with these two restriction enzymes and then inserted into the plasmid. The Prom–*Actin*::*GFP::ATG8f* construct was extracted from the pEZS-CL vector with the *Not*I restriction enzyme, which generates blunt ends. This construct was finally inserted in the pCAMBIA vector (Cambia, Canberra, Australia) previously digested with *Sma*I.

Plant transformations were performed via *Agrobacterium tumefaciens* (GV3101::pMP90) using the floral dipping technique (Clough and Bent, 1998).

### Silique and seed developmental stages

Determination of silique and seed developmental stages was performed according to Baud *et al.* (2008). Just before the opening of the floral bud, a cotton thread was tied around the bud pedicle. This moment coincides with the pollination step and was used as a reference for the age determination of siliques and seeds. The developmental unit used in this study is days after fertilization (DAF).

### RNA isolation

When harvested, Arabidopsis siliques were immediately frozen in liquid nitrogen, then stored at –80 °C. For RNA isolation, three frozen siliques were ground using a mortar and pestle previously cooled with liquid nitrogen. In order to avoid the formation of a viscous paste, 250 µl of extraction buffer (0.1 M LiCl, 0.1 M Tris pH 8, 10 mM EDTA, 1% SDS, 1.5% β-mercaptoethanol) was added to the mortar during grinding. The powder obtained was treated twice with 450 µl of phenol/chloroform 5:1 (P1944, Sigma), vortexed, and centrifuged (13 000 *g*, 15 min, 4 °C). The supernatant phase was transferred to a 2-ml Eppendorf tube and 400 µl of 8 M LiCl solution was added and slowly mixed by turning the tube several times. RNAs were precipitated for 1 h at –80 °C and the tube was then centrifuged (13 000 *g*, 30 min, 4 °C). Then, 1 ml of 70% ethanol was added to the tube and it was centrifuged again (13 000 *g*, 30 min, 4 °C). The supernatant phase was removed and the pellet containing the RNA was dried by pipetting. Dry pellets were rehydrated with 40 µl of RNase-free water (average yield: 1 µg µl^–1^). After extraction, RNAs were purified using a Micro Bio-Spin column (7326206, Bio-Rad). Insoluble polyvinylpolypyrrolidone (PVPP, 5 mg) was placed on the column and then 40 µl of previously extracted RNA was added. The column was placed in an open 2-mL Eppendorf tube and centrifuged (1000 *g*, 2 min, room temperature). The collected RNAs were purified four times through the column (average yield: 0.5 µg µl^–1^), and then 2 µg of RNA was treated with the DNase I, RNase-free kit (EN0521, ThermoFisher Scientific). An inhibitor of RNase, RiboLock RNase Inhibitor (E00382, ThermoFisher Scientific) was added to the reaction mixture according to the manufacturer’s protocol.

### Reverse transcription and quantitative RT-qPCR

Reverse transcription of extracted RNA into cDNA was realized using the M-MuLV Reverse Transcriptase kit (EP0352, ThermoFisher Scientific) according to the manufacturer’s protocol.

The 20-µl reaction mixture contained 5 µl of cDNA (corresponding to 25 × 10^−2^ to 25 × 10^−5^ µg of cDNA), 0.6 µl of each primer (10 µM), 10 µl of a Takyon Rox SYBR MasterMix dTT Blue solution (UF-RSMT-B0710, Eurogentec, Liège, Belgium) containing the Taq polymerase, the dNTPs, and the Sybr Green in a reaction buffer, and 3.8 µl of water.

The RT-qPCRs were run on a CFX 96 thermocycler (Biorad) using a first step at 95 °C for 5 min and then 40 cycles of 5 s at 95 °C, 20 s at 60 °C, and 20 s at 72 °C. A final step consisted in an increase of 0.1 °C s^–1^ to 95 °C.

The primers used for RT-qPCR had an average length of 20 bases and were designed in order to amplify fragments between 100 and 240 bp (see Supplementary Table S1). All primers presented an efficiency of 100 ± 5%. For all RT-qPCR analyses, *EF-1α* (At5g60390) and *APC2* (At2g04660) were used as reference genes (Dekkers *et al.*, 2012; Srivastava *et al.*, 2017).

### Tissue fixation

Harvested siliques were opened and immediately fixed under vacuum with a formaldehyde solution (4% w/v paraformaldehyde in PBS buffer; 10 mM NA_2_HPO_4_, 150 mM NaCl, pH 7,2) overnight at 4 °C.

### Seed discolouration with sodium hydroxide

Fixed seeds were discoloured for at least 2 h at 37 °C in a sodium hydroxide solution (NaOH 200 mM, SDS 1%) then rinsed twice in water for 5 min. At this step, young seeds became pink due to the oxidation of tannins. Seeds were then discoloured for 30 min in a 5% bleach solution, then rinsed twice in water for 5 min. This discolouration technique was used on seeds after GUS staining.

### GUS staining of seeds and siliques

Siliques were harvested, fixed and then incubated in a freshly prepared infiltration buffer (50 mM pH 7 phosphate buffer, 10 mM EDTA, 1% Triton X-100, 2 mM X-GlucA). After infiltration under vacuum, samples were placed in the dark at 37 °C for 2–24 h depending on the tissue. After incubation, samples were embedded in a resin, or directly observed, or discoloured by sodium hydroxide. White-light imaging was performed using a DMRB microscope (Leica).

### Sample sections of siliques

For embedding of silique in resin or wax, samples have to be dehydrated in ethanol first. Fixed siliques were therefore immersed successively in solutions with an increasing gradient of ethanol (30%, 50%, 70%, and 100%, completed with PBS buffer) for at least 1 h for each solution.

### Resin embedding of siliques

After GUS staining, dehydrated siliques were immersed successively in solutions with an increasing gradient of the Technovit 7100 kit resin (14655, VWR International, Radnor, PA, USA), namely 30%, 50%, 70%, and 100% resin completed with ethanol, for 12–24 hours for each solution. At the last step, samples had to stay in pure resin for at least 5 d. Then, siliques were placed in silicone moulds. A Hardener II solution provided with the kit was added to the resin and poured into the moulds. When they became hard, the blocks of resin were taken out of the moulds and silique sections with a thickness of 10 µm were taken using a rotating microtome (Leica).

### Wax embedding of siliques

Wax embedding was used before immunolocalization on slides. After fixation and dehydration, siliques were immersed in a solution containing 50% wax (polyethylene glycol/1-hexadecanol 9:1 w/v) and 50% ethanol for 12 h at 40 °C, and then three times in a solution of 100% wax for 12 h each at 40 °C. Siliques were then placed in silicone moulds and covered with pure wax. When the wax became hard at room temperature, the blocks were taken out of the moulds and silique sections with a thickness of 10 µm were taken using a rotating microtome (Leica).

### Immunolocalization of autophagosomes

Immunolocalization was carried out on siliques from plants expressing the Prom–*Actin*::*GFP*::*ATG8f* construct. Siliques were embedded in wax and cut using a microtome, then the sections were placed on Polysine slides (P4981, ThermoFisher Scientific). The slides were then successively immersed twice in ethanol for 10 min, in a solution of 50% ethanol and 50% PBS for 5 min, in a solution of 30% ethanol and 70% PBS for 5 min, and twice in a PBS solution for 5 min. When the wax was removed, the slides were immersed twice in a citrate buffer (4261, Promega) for 1 min at 90 °C and rinsed in PBS buffer for 5 min. The slides were then immersed in a bovine serum albumin solution (PBS, BSA 1%) for 1 h at room temperature. Next, 500 µl of 1/500 diluted primary anti-GFP monoclonal antibody (Clontech) produced in mice was deposited on the slides, which were then incubated overnight at 4 °C in a humid compartment. The slides were rinsed three times in PBS for 10 min before the addition of 500 µl of 1/250 diluted secondary antibody, Alexa Fluor 488 (ThermoFisher Scientific) produced in goat. A coverslip was placed on each slide with a solution composed of 50% glycerol and 50% PBS. Confocal observations were carried out using a TCS SP5 microscope (Leica).

### Seed discolouration with chloral hydrate

In order to observe embryo development, siliques from 2–12 DAF were opened and the seeds were discoloured for 2–24 h in a chloral/glycerol/water solution (8:1:2, w/v/v) (Xu *et al.*, 2016). Discoloured seeds were then placed between a slide and coverslip and were observed using a Nomarski objective (DIC) on an AxioObserver microscope (Zeiss).

### Quantification of total proteins

During harvesting, siliques were opened and seeds removed from the valves. Developing seeds were then frozen in liquid nitrogen and lyophilized. Then, 2 mg of dry seeds was ground using a ball mill with 50 µl of an extraction buffer [50 mM pH 7.5 Hepes, 3% SDS, 10% glycerol, 150 mM NaCl, 1× protease inhibitor (cOmplete Protease Inhibitor Cocktail; 11697498001, Roche), 0.1 M proteasome inhibitor MG132 (M7449, Sigma), 20 mM DTT). Ground seeds were then centrifuged (13 000 *g*, 15 min, room temperature) and 10 µl of the supernatant was used for total protein quantification using the 2D-Quant kit (GE80-6483-56, GE Healthcare, Little Chalfont, UK) according to the manufacturer’s protocol.

### SDS-PAGE electrophoresis

Protein extraction was performed from 2 mg of dry seeds for each sample according to the protocol used by Deruyffelaere *et al.* (2015). Protein separation, Coomassie colouration, and western blots were also carried out according to Deruyffelaere *et al.* (2015).

Polyclonal 12S globulin and 2S albumin sera were kindly provided by Sabine d'Andréa and used as described in d'Andréa *et al.* (2007). Quantification of protein bands was performed using the ImageJ software (https://imagej.nih.gov/ij/).

## Results

### Expression of *ATG* genes increases during silique development

The expression level of the 34 *ATG*-related genes listed in Supplementary Table S1 was monitored during seed development on siliques (pericarp plus seeds) harvested at 4, 12, 20, and 28 d after flowering (DAF).

**Fig. 1.**
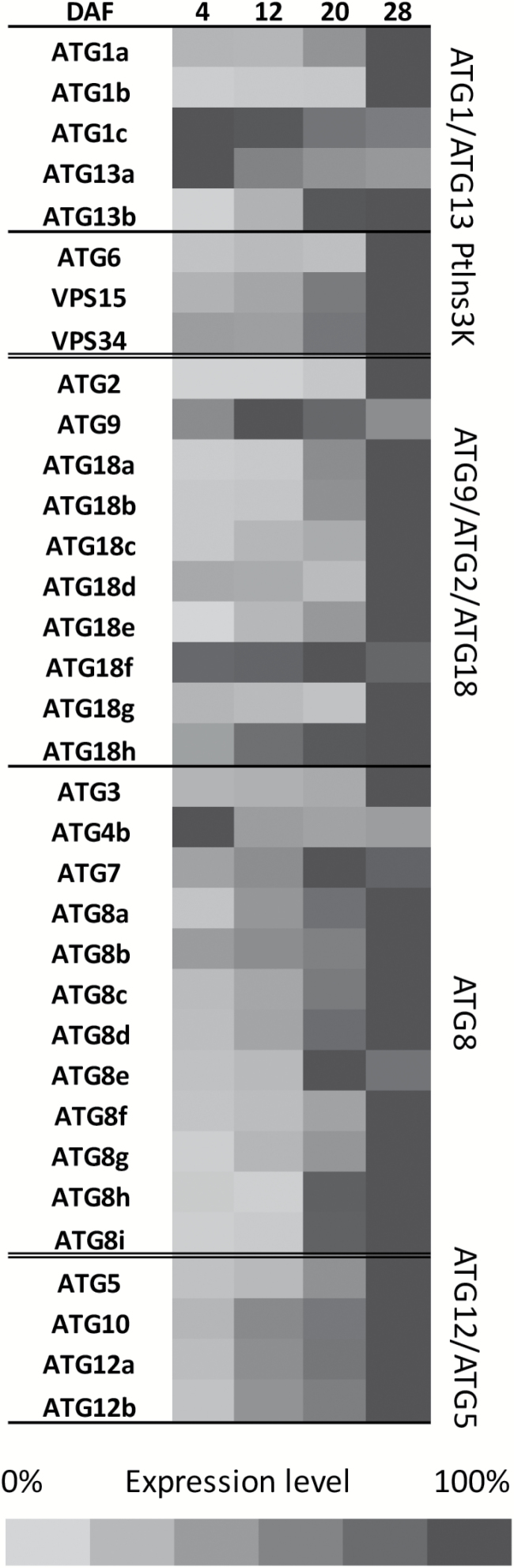
Relative expression level of Arabidopsis *ATG* genes in whole siliques during development. Functional ATG groups are shown: ATG1/ATG13 induce the autophagosome formation, PtIns3K and ATG9/ATG2/ATG18 are involved in the formation of pre-autophagosomal structures and lipid recruitment, and the ATG8 and ATG12/ATG5 conjugation systems enlarge and close the autophagosome. Gene expression levels were monitored by RT-qPCR using specific primers (Supplementary Table S1) and normalized to a synthetic reference gene that combines *EF-1α* and *APC2*, taking the reference *C*t value as √(*EF*-1α × *APC2*). Gene expressions were then normalized to the highest value of each gene and are displayed according to the scale bar. Data are the means of the expression values measured on siliques of three plants from three different plant cultures. Genes clustering was performed using XLSTAT. DAF, days after fertilization.

The relative expression of almost all the *ATG* genes increased during seed development and especially during maturation (after 12 DAF; Fig. 1). Only three genes showed decreased expression during silique development, namely *ATG1c* and *ATG13a* that belong to the AT1/ATG13 complex, which plays a role in the induction of the autophagy process, and *ATG4b* that is involved in the ATG8 conjugation system. The majority of the genes participating in the ATG8 conjugation system were expressed early compared with the other functional ATG groups, indicating that this system could be involved in another process. In the ATG9 complex involving ATG2 and ATG18, *ATG9* showed optimal expression levels at 12 DAF and *ATG18f* and *ATG18h*, as well as *ATG7* and *ATG8e* in the ATG8 complex, optimal expression levels at 20 DAF.

The data suggested that autophagy was clearly induced from the early stage of silique development and that it increased sharply after 20 DAF.

### Tissue-specific expression of *ATG8f* genes in siliques and seed

**Fig. 2.**
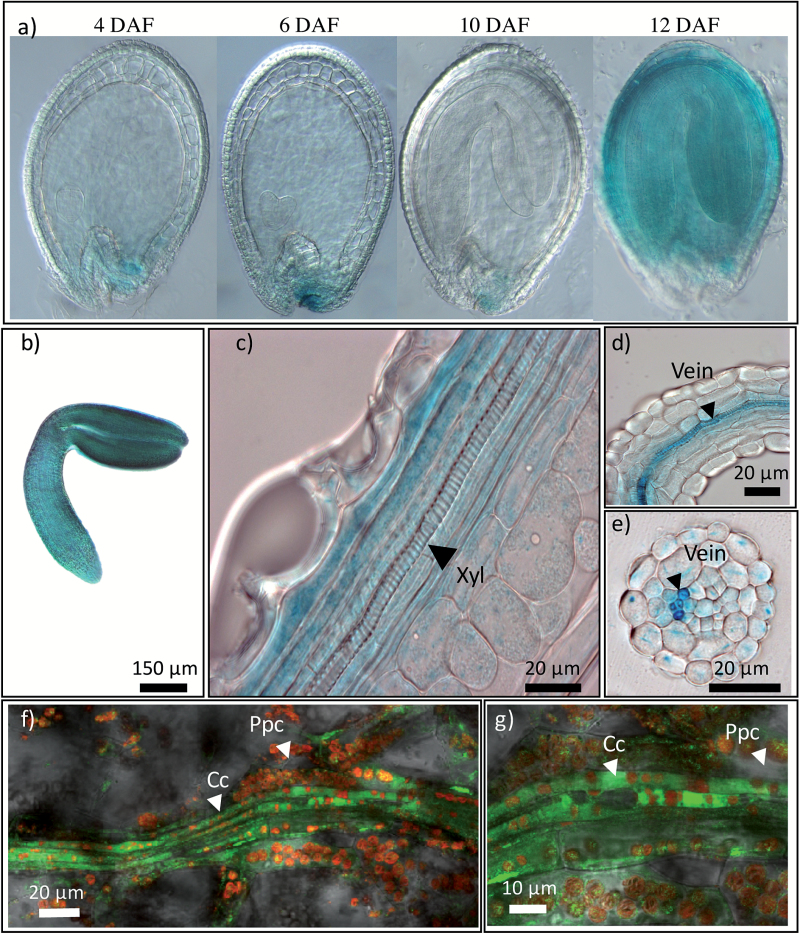
*ATG8f* is expressed in the veins of the pericarp and in the seed embryo. Localization of *ATG8f* expression was observed after GUS staining of Prom–*ATG8f::UIDA* siliques in (a) seeds, (b) embryo (12 DAF), (c) pericarp (12 DAF), and (d, e) funiculus veins (12 DAF). (f, g) GFP fluorescence (green) in pericarps expressing the Prom–*ATG8f::GFP* construct observed at 12 DAF and chloroplast autofluorescence (red) show that Prom–*ATG8f* is mainly expressed in companion cells. For each construct, eight plants were observed. Cc, companion cells; Ppc, phloem parenchyma; Xyl, xylem. DAF, days after fertilization.

The expression pattern of several *ATG8* genes was then investigated using Prom–*ATG8::UIDA* fusions in pericarps and in seeds. GUS staining in the seeds at the globular (4 DAF), heart (6 DAF), bent (10 DAF), and mature (12 DAF) stages was more or less intense depending on the promoters and constructs used. Thus, we observed that all the Prom–*ATG8::UIDA* fusions were expressed mostly in the chalaza from the globular stage onwards, and in the embryo at different stages (Fig. 2a and Supplementary Fig. S1). The most spectacular GUS staining was observed with the *ATG8f* promoter, which developed an intense blue signal in embryos of mature green seeds (Fig. 2a, b and Supplementary Fig. S1). In the pericarp, the Prom–*ATG8f::UIDA* GUS staining was mainly detected in the vasculature of the pericarps (Fig. 2c) and funiculus (Fig. 2d, e). Transgenic plants carrying Prom–*ATG8f::GFP* constructs confirmed promoter activity in the veins of the pericarp and showed that it was restricted to the phloem companion cells and phloem parenchyma cells (Fig. 2f, g). Thus, a typical single-file alignment of autofluorescent chloroplasts was detected in companion cells, whereas the chloroplasts were located at the cell periphery of the phloem parenchyma tissue (Cayla *et al.*, 2015).

### Visualization of autophagosomes in embryos

**Fig. 3.**
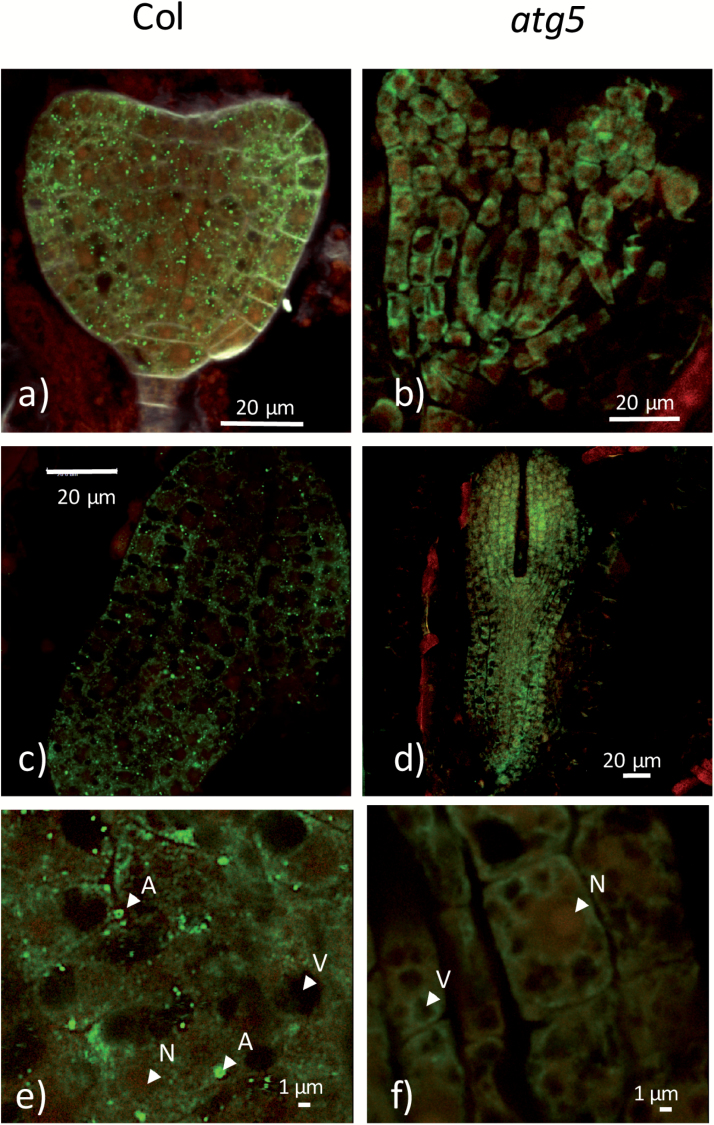
Autophagosomes are observed in embryos. Immunolocalization of the GFP::ATG8f protein fusion in (a, b) heart embryos (6 DAF) and (c–f) torpedo embryos (8 DAF) expressing Prom–*Actin::GFP::ATG8f* revealed the presence of autophagosomes in the Col wild-type (a, c, e) but not in the *atg5* mutant (b, d, f). Cross-sections of the embryos were incubated with GFP monoclonal antibodies and then with the Alexa Fluor 488 secondary antibody. Fluorescent signals of the labelled GFP::ATG8f (shown as green dots) were observed by confocal microscopy on eight plants. Tissue autofluorescence is in red. A, autophagosome; N, nucleus; V, vacuole. Controls with the Alexa Fluor 488 secondary antibody alone are shown in Supplementary Fig. S2. DAF, days after fertilization.

Many attempts to visualize autophagosomes in seeds were tried using confocal microscopy on fresh or dry seeds using the Prom–*35S*::*GFP*::*ATG8*, Prom–*Actin*::*GFP*::*ATG8*, and Prom–*Ubi*::*GFP*::*ATG8* transformants that have been previously used successfully for observations on roots or leaves in our laboratory. Unfortunately, however, GFP fluorescence observations on seed material proved to be difficult and not convincing. Therefore, seed material was embedded and autophagosome structures were observed through GFP immunolocalization performed on the seeds of both WT and *atg5* transgenics harbouring the Prom–*Actin*::*GFP*::*ATG8f* construct. Immunolocalization with an anti-GFP antibody and using the Alexa Fluor 488 secondary antibody revealed many fluorescent dots in the WT background (Fig. 3a, c, e) that were absent in *atg5* background (Fig. 3b, d, f). The size of the fluorescent structures was less than 1 µm (Fig. 3e), which is in good agreement with the size of autophagosomes or pre-autophagosomal structures. The negative controls with the secondary antibody alone are shown in Supplementary Fig. S2.

### *atg5* mutant seeds exhibit abortion and early browning

**Fig. 4.**
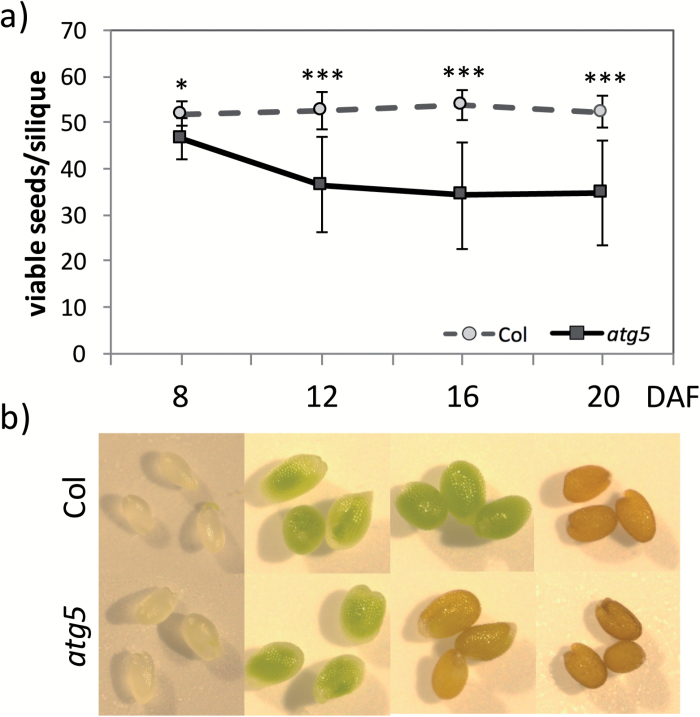
Browning phenotype of *atg5* seeds of plants grown under high-nitrate conditions. (a) The number of fully developed viable seeds per silique was monitored during development in the wild-type (Col) and the *atg5* mutant. Means (±SD) of 20 siliques are shown. *P <0.05, ***P<0.001. DAF, days after fertilization. (b) Imaging of *atg5* and WT seeds, using the same magnification throughout. Similar results were obtained on seeds from plants grown with low nitrate and are presented in Supplementary Fig. S4.

Seed abortion was monitored by counting the viable fully-developed seeds in siliques. Because nutrition of the mother plant impacts significantly on metabolism and seed production in autophagy mutants, analyses were performed on plants grown under low- (2 mM) and high- (10 mM) nitrate conditions (see phenotypes of the Col and *atg5* rosettes in Supplementary Fig. S3). A strong difference was observed between mutants and the WT for seed viability and seed colour, but no significant differences were detected between the two nitrate regimes (Fig. 4 and Supplementary Fig. S4, showing 10 mM and 2 mM, respectively). Significantly lower seed viability was observed as early as 12 DAF in *atg5* relative to Col (21% and 33% of seed abortion in *atg5* under high- and low-nitrate conditions, respectively). Low-nitrate conditions also increased seed abortion in Col (from 0 to 6%). In *atg5*, seed abortion was not systematically observed in all siliques. We also observed that *atg5* seeds turned brownish from 16 DAF onwards, while Col seeds remained green longer and turned brown only at the end of their development, i.e. from 20 DAF (Fig. 4b and Supplementary Fig. S4b, showing 10 mM and 2 mM, respectively).

**Fig. 5.**
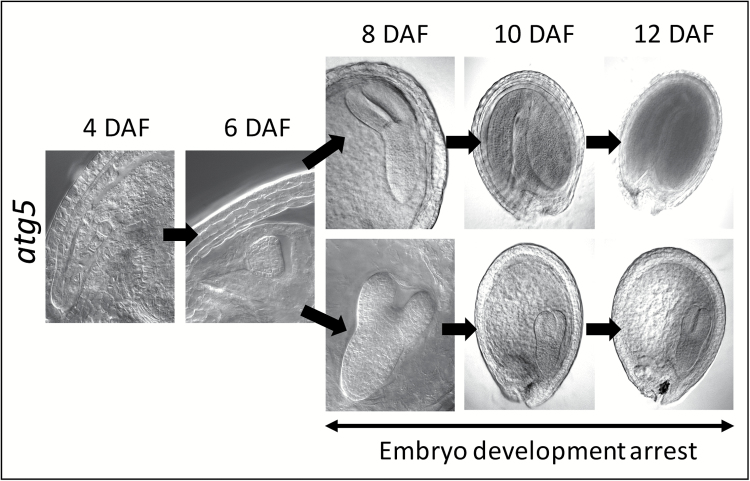
Embryo development is arrested in the *atg5* mutant. Observation of *atg5* embryos from plants grown under high-nitrate conditions showed both wild type-like and aborted phenotypes after 8 DAF. Seeds were treated with choral hydrate before observation with Nomarski optics. DAF, days after fertilization.

Observation of embryos after chloral hydrate treatment and using Nomarski optics revealed that arrest of development occurred between 8 and 12 DAF, mainly at the ‘torpedo’ stage (Fig. 5). The *atg5* embryo presented stocky cotyledons compared to the wild-type.

### *atg5* mutant seeds have altered storage protein accumulation

#### *Low seed weight of the* atg5 *mutant after 20 DAF*

**Fig. 6.**
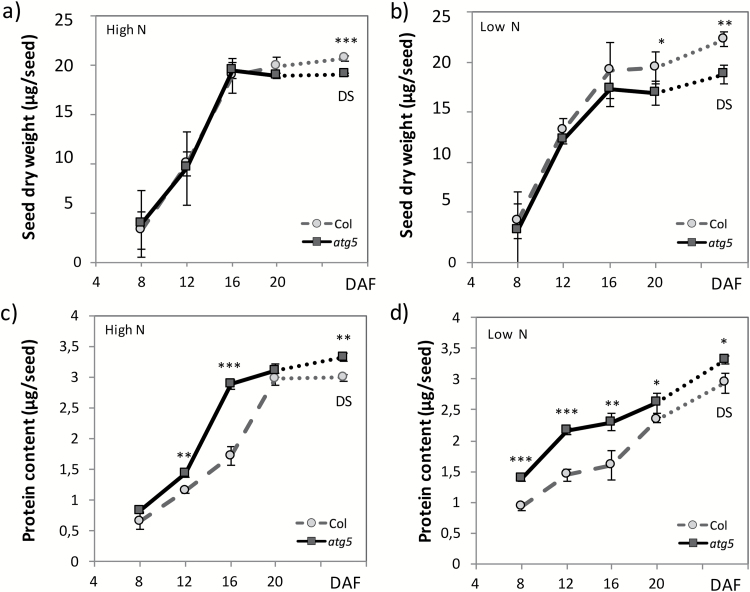
Biomass and protein contents of seeds during development. (a, b) Dry weight and (c, d) total protein content in wild-type Col and *atg5* seeds from plants grown under high- (a, c) or low- (b, d) nitrate conditions. Means (±SD) of three biological replicates are shown. *P<0.05, **P<0.01, ***P<0.001. DAF,: days after fertilization. The dotted lines join the last harvested stage at maturation (20 DAF) to the dry seed (DS) stage. Similar results were obtained for dry weight of *atg7*, *atg4a-4b*, and *sid2-atg5* seeds and are presented in Supplementary Fig. S5.

Only viable seeds were considered and selected for further analyses. There was no significant difference in dry weight (DW) between *atg5* and Col seeds during development until after 20 DAF under high-nitrate conditions, and until after 16 DAF under low-nitrate conditions (Fig. 6a, b). At 20 DAF, when grown under low nitrate, the dry weight of *atg5* seed was slightly lower than that of Col seed. At maturity, the dry seed (DS) of *atg5* was significantly lighter than that of Col seed (Fig. 6a, b) in both nutritional conditions. The single *atg7* and the double *atg4a-4b* mutants also produced dry seed with lower weight than the wild-type (Supplementary Fig. S5).

#### *High and early accumulation of total protein in the* atg5 *mutant*

There was no significant difference in DW between *atg5* and Col seeds until 20 DAF, meaning that protein content per seed was a good indicator of protein concentrations at these stages. Higher protein contents were measured in *atg5* seeds relative to Col seeds (Fig. 6c, d), and differences were significant under both low and high nitrate, except at 8 and 20 DAF under high nitrate (Fig. 6c). This indicated that protein accumulation was globally higher and occurred earlier in the *atg5* mutant than in the WT.

#### *Increased ratio of high/low molecular-weight of total proteins in the* atg5 *mutant*

**Fig. 7.**
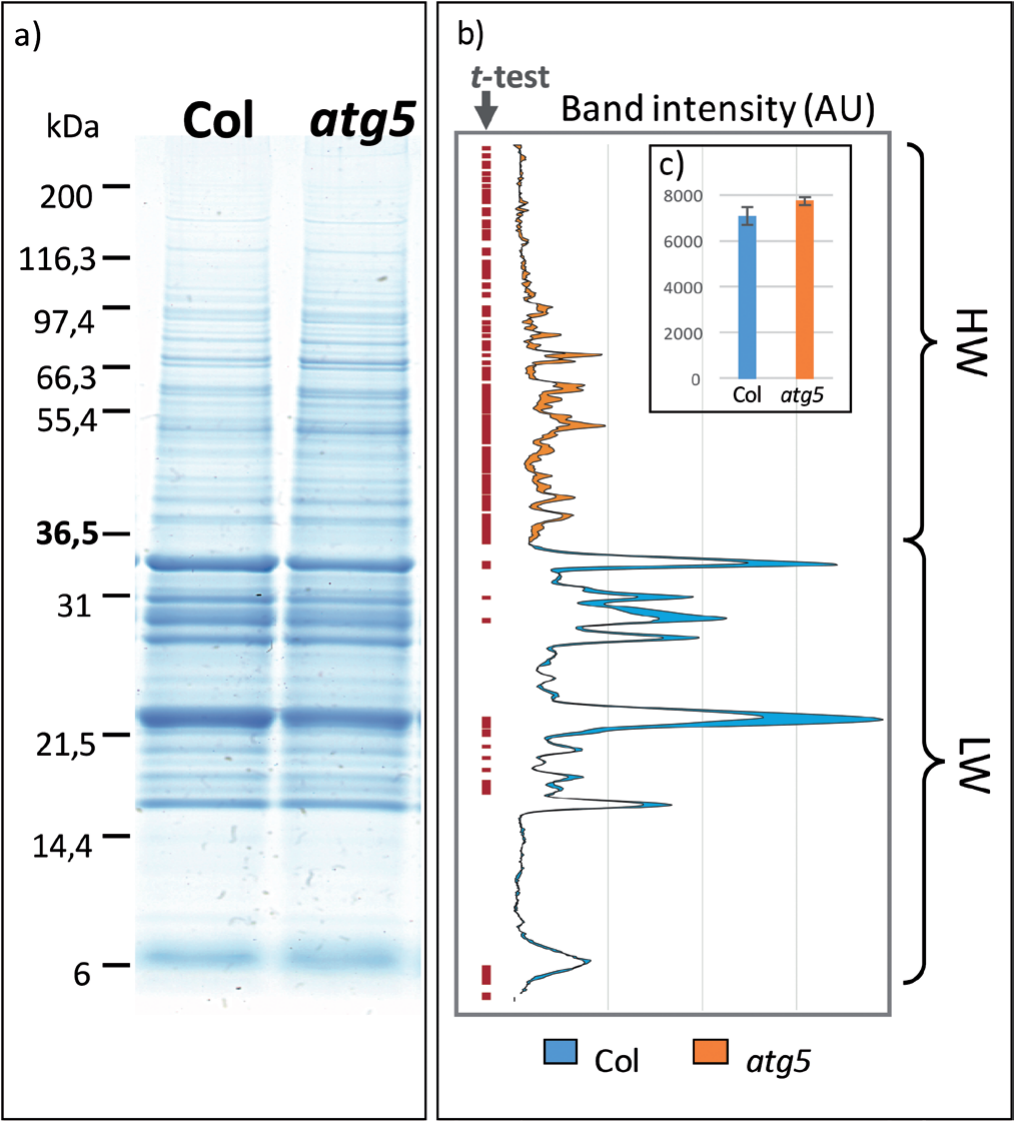
Protein profiles of wild-type Col and *atg5* dry seeds produced under high-nitrate conditions. Total proteins extracted from dry seeds were separated on SDS-PAGE gels and stained with Coomassie Blue (a). The same quantity of protein was loaded in each lane. The graphs in (b) represent the average of the band intensities (AU, arbitrary units) measured on three biological repeats. Significant differences between Col and *atg5* are indicated by the red dots on the left (*t*-test, *P*<0.05). Differences between Col and *atg5* protein contents are presented in orange when the content was higher in *atg5* than in Col, and in blue when the content was higher in Col than in *atg5*. The orange and blue colours correspond to the high-weight (HW, >37 kDa) and low-weight (LW, <37 kDa) proteins, respectively. The sum of the band intensities in the Col and *atg5* protein extracts is indicated in (c) as the loading control. Similar results were obtained on seeds from plants grown with low nitrate and are presented in Supplementary Fig. S6.

In order to distinguish the nature of the proteins accumulated in *atg5* seeds, protein profiles of dry seeds were examined on Coomassie Blue-stained SDS-PAGE gels (Fig. 7 and Supplementary Fig. S6). The differences in profiles between *atg5* and Col were similar under both high- (Fig. 7) and low- (Supplementary Fig. S6) nitrate conditions, as attested by quantification of the band intensities performed after gel scanning. The results showed that high molecular-weight proteins (from 200 to 37 kDa) were significantly more abundant in *atg5* than in Col seeds, while in contrast, low molecular-weight proteins, mainly representing globulin and albumin, were significantly less abundant in *atg5* than in Col seeds. The single *atg7* and the double *atg4a-4b* mutants presented the same profile in Coomassie gels, with around 30% of high-weight (HW) proteins compared to 20% of HW in the wild-type (Supplementary Fig. S7). The ratio of HW/LW for the *sid2-atg5* double-mutant was also higher compared to those of *sid2* (Supplementary Fig. S7).

#### *Low and early 12S globulin content in the* atg5 *mutant*

**Fig. 8.**
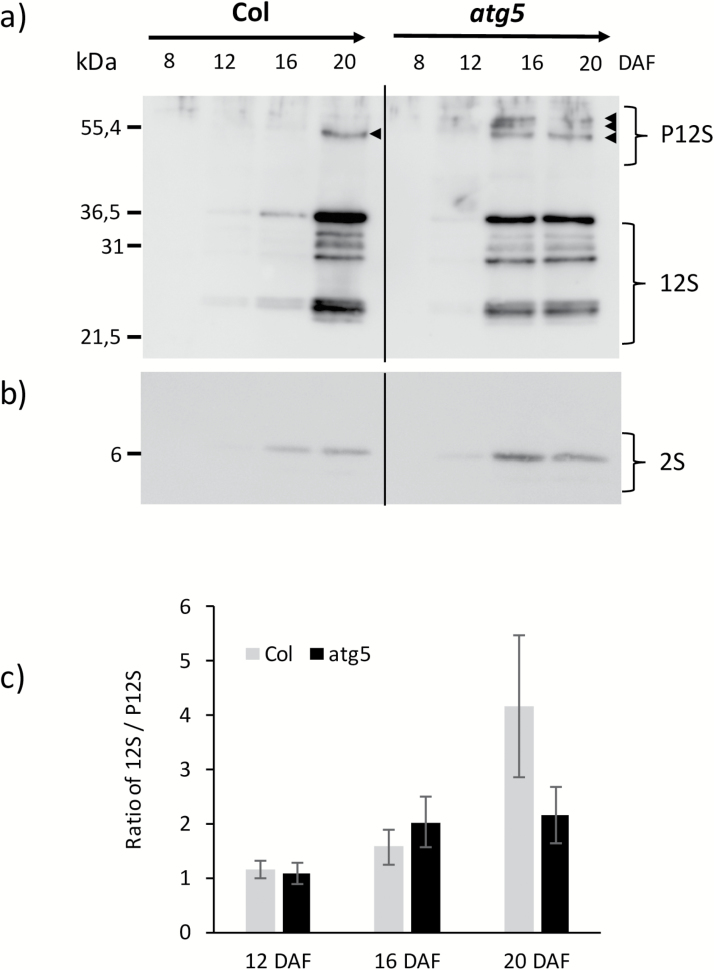
Kinetics of 12S and 2S protein accumulation in wild-type Col and the *atg5* mutant during seed development. Seeds were obtained from plants grown under high-nitrate conditions. Western blots using (a) 12S and (b) 2S storage protein antibodies are shown. Total proteins extracted from the same number of seeds were loaded in each lane. Ratios of the 12S proteins to 12S precursors (c) were determined using ImageJ software. Means (±SD) are shown (*n*=3). DAF, days after fertilization; P12S, precursors of 12S proteins (indicated by arrows).

Antibodies raised against the 12S globulins and 2S albumins were used to quantify the accumulation of the storage proteins during seed development. The kinetics of 12S and 2S accumulation in seeds of *atg5* and Col plants, evaluated on western blots, showed that proteins, mostly 12S, accumulated earlier in *atg5* seeds than in Col seeds (Fig. 8a). Signals for the 12S, 2S, and 12S-precursors were clearly observed at 16 DAF in *atg5* seed extracts, but were only very slightly visible in Col seed extracts (Fig. 8a, b). At 20 DAF, 12S storage proteins appeared less abundant in *atg5* seeds than in Col seeds, while 12S-precursors appeared as several distinct bands that were absent from Col seed extracts (Fig. 8a). The ratio of 12S to 12S-precursors increased between 16 and 20 DAF in the Col seeds but remained unchanged in the *atg5* seeds (Fig. 8c).

**Fig. 9.**
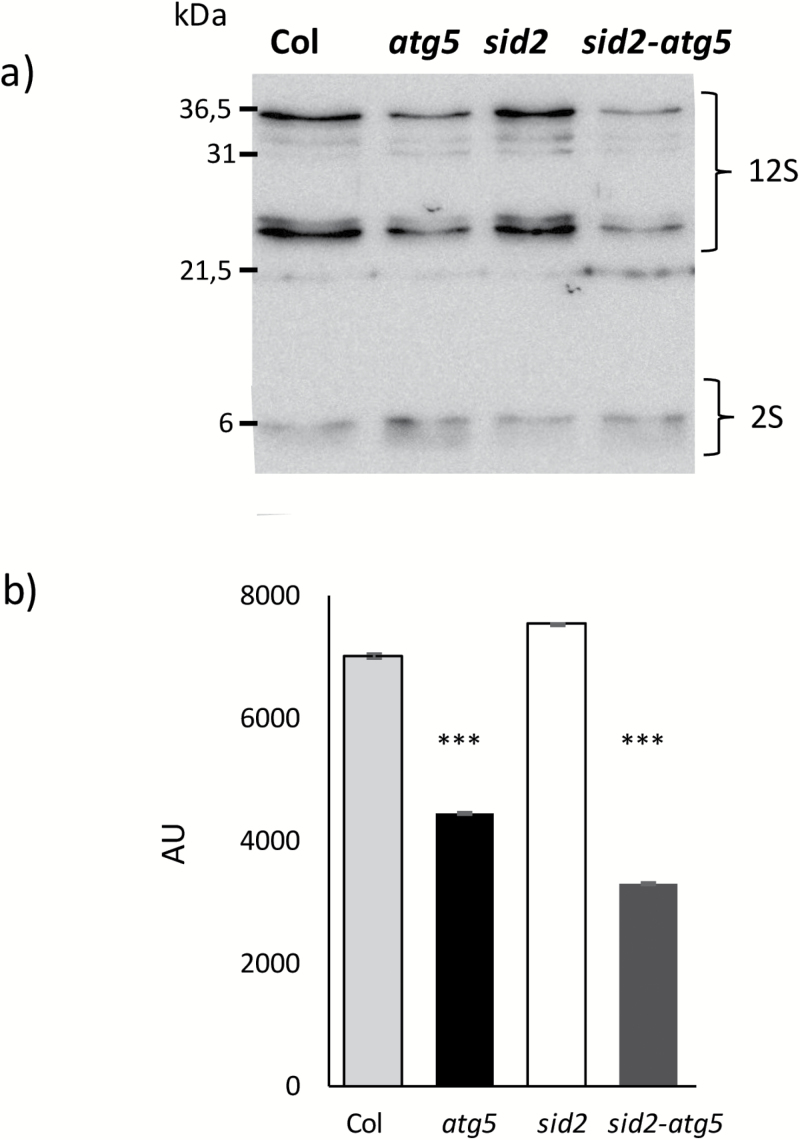
12S globulin and 2S albumin protein contents in dry seeds. Seeds were obtained from wild-type Col, *atg5*, *sid2*, and *sid2-atg5* plants grown under low-nitrate conditions. (a) Western blots were performed using 12S and 2S storage protein antibodies. (b) The means (±SD; *n*=3) of the band intensities quantified with ImageJ software (AU, arbitrary units) of 12S proteins in dry seeds of Col, *atg5*, *sid2*, and *sid2-atg5*. Protein loading was performed on the basis of the same number of dry seeds. ***P<0.001. Similar results were obtained on the dry seeds of *atg7* and *atg4a-4b* mutants and are presented in Supplementary Fig. S8.

The lower 12S globulin content in *atg5* dry seeds relative to Col dry seeds was also observed in the *sid2-atg5* double-mutant compared to the *sid2* mutant (Fig. 9a, b). Nitrate nutrition had no effect on the accumulation of storage protein, as the difference between Col and *atg5* was observed under high- and low-nitrate conditions (Supplementary Fig. S8a,b). In addition, the lower 12S globulin content compared to Col was also observed in the *atg7* and *atg4a-4b* mutants (Supplementary Fig. S8c,d).

## Discussion

In this study, we show that autophagy gene expression increases during seed development, especially in the embryo, and during the maturation phase that corresponds to the formation of the oil and protein bodies in the embryo (Baud *et al.*, 2008). At earlier developmental stages, all the *ATG8* genes were moderately expressed in the embryo and more strongly in the chalaza, which corresponds to the location where amino acids are released from the phloem, at the end of the funiculus vasculature, through the UmamiT efflux systems (Müller *et al.*, 2015; Tegeder and Masclaux-Daubresse, 2018). Interestingly, the Prom–*ATG8f*::*UIDA* fusion showed a strong expression in the phloem companion cells of the pericarp and in the vasculature of the funiculus. As *ATG* gene expression is a preliminary to autophagy activity (Bernard *et al.*, 2015), finding *ATG8f* promoter activity in the pericarp, funiculus, and chalaza maternal tissues suggests that autophagy plays a role in the nutrient import into the seeds, for development as well as for nutrient storage (Zhang *et al.*, 2007). The strong expression in the zygote embryo at maturity also suggests a role of autophagy in the accumulation of protein and/or lipid reserves.

Although it was not possible to investigate the role of autophagy separately in maternal and zygote tissues, the study of seed features during development in the *atg5* mutant, and the use of different nitrate regimes to grow the plants, provided some clues to this question. Following seed formation on plants grown under low- and high-nitrate conditions, we observed that in both situations early (before 12 DAF) abortion in *atg5* was quite high. This phenotype was not nitrate-dependent but it may be explained by a defect in the capacity of mother tissues to drive nutrients to the seeds. We then presume that autophagy defects in phloem companion cells impaired seed nutrient capture, thus resulting in the seed abortion.

Although viable seeds could be obtained in *atg5*, they were different from the WT. Differences were visible early during development and, as a first symptom, we detected that chlorophyll decay and seed browning appeared earlier, as if seed development was accelerated. Development acceleration was confirmed by the earlier protein storage deposition in *atg5* viable seeds.

As the *atg5* mutant, as well as all the *atg* mutants, exhibited an early senescence that could interfere with the autophagy process, the *sid2-atg5* double-mutant, was used to obtain plants with longer life. The phenotype of this mutant, i.e. low 12S storage protein production, was comparable to that of *atg5*, showing that the observed results were due to the lack of autophagy and not to an early senescence.

The *atg5* viable seeds did not seem to suffer from nitrogen starvation as might have been expected. As reported by Guiboileau *et al.* (2012), we indeed found that the percentage N content in seeds was higher in *atg5* than in the WT (data not shown), and although seed DW was similar in *atg5* and the WT until 16 DAF, seed protein concentration was higher in *atg5* relative to the WT. This feature was nitrate independent. All these results then suggested that the abortion of seeds certainly reduced the overall sink N demand and made the mother resources sufficient to feed the *atg*5 viable seeds properly.

The higher protein content in *atg5* seeds was not paralleled by an increase in 12S and 2S storage proteins. The reduced production of storage proteins observed in all the *atg* mutants tested was mostly manifested for 12S proteins and to a lesser extent for the 2S. We showed that the higher protein content of the *atg5* mutant seed was probably correlated with higher storage protein precursors. This observation then questions the role of autophagy in the processing of seed storage proteins. Considering all the pathways of storage protein deposition, some key steps could be controlled by the autophagy process. Precursors of 12S globulins are synthesized in the endoplasmic reticulum and Golgi from proforms and transported to the protein storage vacuoles (PSVs) *via* multi-vesicular bodies (MVBs). Then, vacuolar processing enzymes (VPEs, mainly VPEβ) convert the precursors into seed storage protein (Shimada *et al.*, 2003; Otegui *et al.*, 2006). It has been reported that *atg* mutants suffer from oxidative stress (Masclaux-Daubresse *et al.*, 2014) and hence the role of reactive oxygen species throughout the course of storage protein processing should be considered. The accumulation of oxidized proteins and organelle components could potentially affect (i) the maturation of the proforms into 12S precursors, (ii) the transport of the precursors in the MVBs, and/or to the PSVs, and (iii) the processing of precursors into mature storage protein.

Whether autophagy plays a role in storage protein processing is unclear, but this has been suggested in maize by Reyes *et al.* (2011). These authors found that zeins (a class of prolamine proteins) were delivered to aleurone PSVs through pre-vacuolar compartments consisting of multi-layered membranes that suggested autophagosome structures. However, the absence of ATG8 decoration in the membranes of these structures led them to the conclusion that they were not typical autophagosomes. As alterations in these structures has not been verified in the recently obtained maize autophagy mutants (Li *et al.*, 2015), it cannot be said whether they are actually macroautophagy-dependent.

A recent publication from Gao *et al.* (2015) identified the FREE1-ESCRT component as required for MVB biogenesis and protein transport to the PSV. The authors showed a strong defect in storage protein processing in the *free1* mutant, leading to the accumulation of precursors of storage proteins just as we observed in *atg*5 seeds. They also showed that the loss of function of FREE1 disturbed the autophagy pathway, promoting autophagosome–MVB fusion events and decreasing autophagosome fusion to the vacuole.

Our results showed that, like the *free1* mutation, the *atg*5 mutation affects storage protein deposition, and thus that both the FREE1-dependent ESCRT and the autophagy mechanisms are needed for seed storage protein maturation. How these two pathways contribute together to PSV formation needs further investigation. Whether autophagy contributes to storage protein processing that facilitates VPE trafficking to the MVBs or PSVs is a possibility that needs to be considered. Nevertheless, our study describes a new role for autophagy that is distinct from the usual degradation function assigned to this intracellular process up to now.

## Supplementary data

Supplementary data are available at *JXB* online.

Fig. S1. Localization of the expression of *ATG8c*, *e*, *f*, and *g* during embryo development in seeds.

Fig. S2. Autophagosomes observed in embryos.

Fig. S3. Development of Col and *atg5* rosettes.

Fig. S4. Browning phenotype of *atg5* seeds from plants grown under low-nitrate conditions.

Fig. S5. Dry weight of seeds from Col and mutant plants.

Fig. S6. Protein profiles of dry seeds of Col and *atg5* produced under low-nitrate conditions.

Fig. S7. Protein profiles of dry seeds of Col and *atg* mutants produced under low-nitrate conditions.

Fig. S8. 12S globulin and 2S albumin protein contents in dry seeds of Col and *atg* mutants.

Table S1. Primers used for RT-qPCR analyses and cloning.

Supplementary Figure and Table
