# Supplementary material for: Autophagy controls resource allocation and protein storage accumulation in Arabidopsis seeds
Source: J Exp Bot. 2018 Feb 23;69(6):1403–14. doi: 10.1093/jxb/ery012 (PMC6018931; doi:10.1093/jxb/ery012)
Supplement: Supplementary Figure and Table [file ery012_suppl_supplementary_figure_and_table.pdf]

Autophagy controls resource allocations and protein storage accumulation in Arabidopsis seeds

Julien Di Berardino, Anne Marmagne, Adeline Berger, Kohki Yoshimoto, Gwendal Cueff, Fabien Chardon, Céline Masclaux-Daubresse and Michèle Reisdorf-Cren

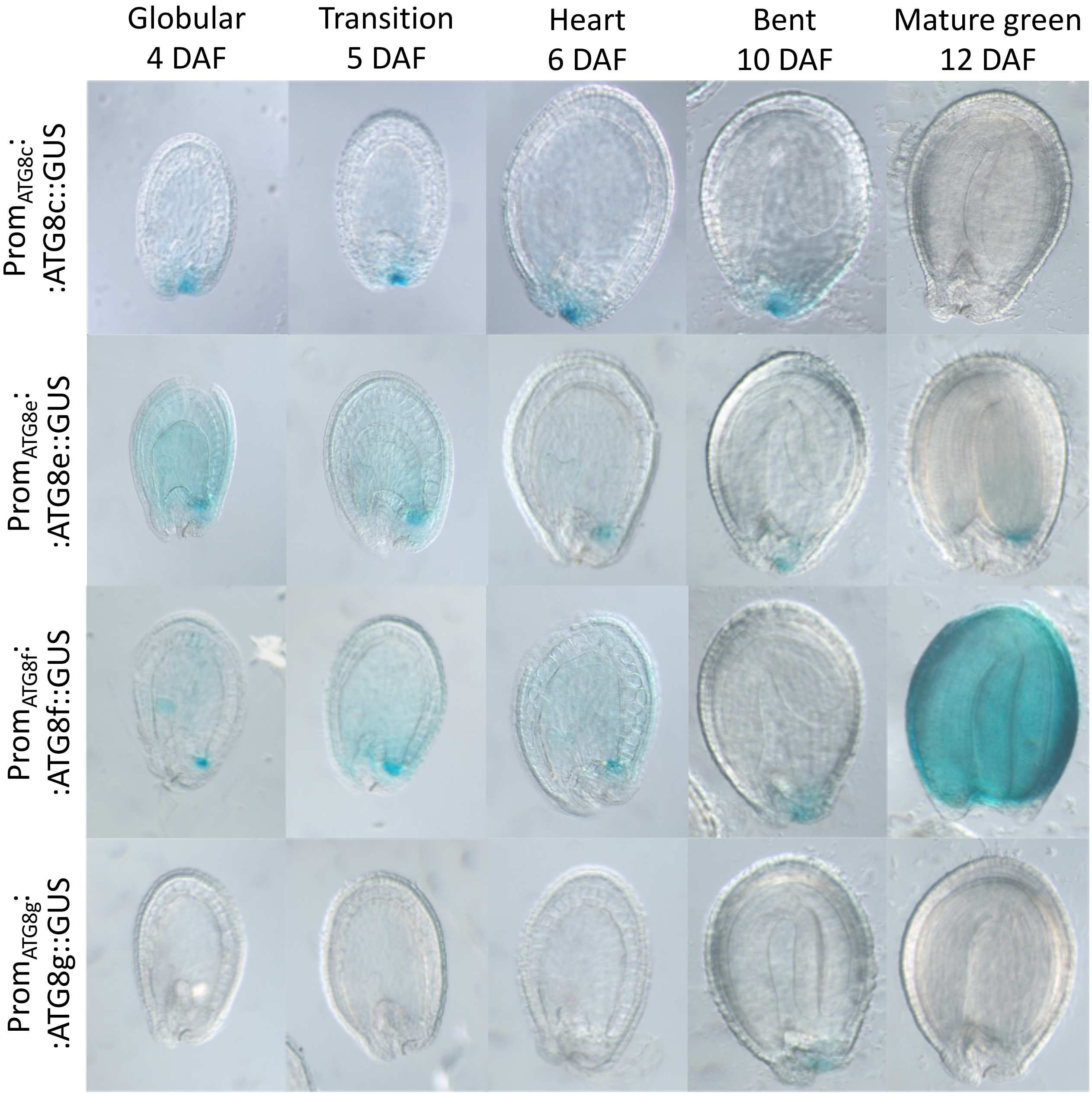

Supplementary Fig. S1. Localization of the expression of *ATG8c, e, f* and *g* during embryo development in seeds. Localization was observed after GUS staining of Prom-*ATG8*::*ATG8*::*UIDA* plants (Slávikovà *et al*. 2005). Imaging of seeds was performed using the same magnification. Eight plants were observed for each construct and each developmental stage. DAF: Days After Fertilization.

Autophagy controls resource allocations and protein storage accumulation in Arabidopsis seeds

Julien Di Berardino, Anne Marmagne, Adeline Berger, Kohki Yoshimoto, Gwendal Cueff, Fabien Chardon, Céline Masclaux-Daubresse and Michèle Reisdorf-Cren

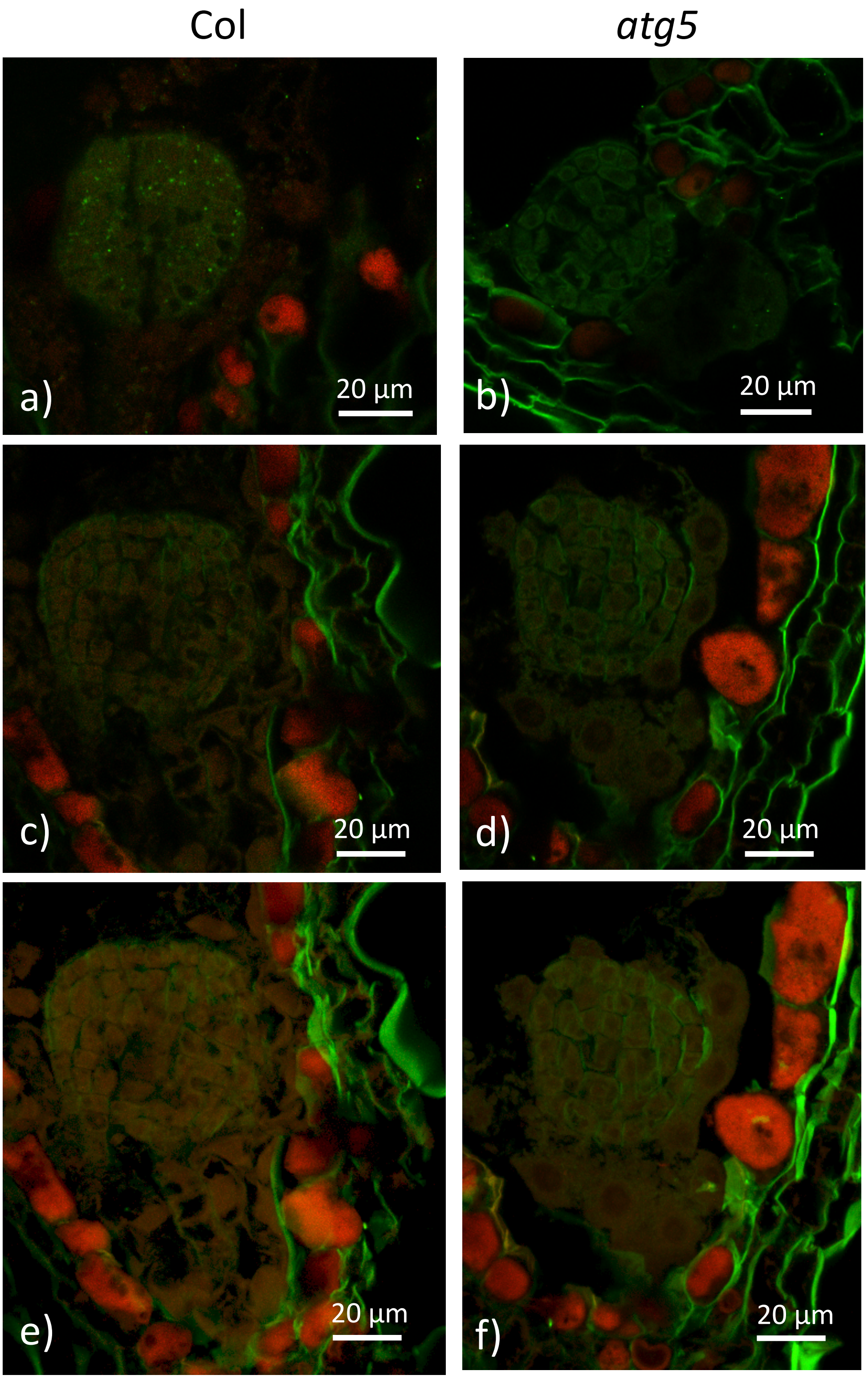

Supplementary Fig. S2. Autophagosomes are observed in embryos. Immunolocalization of the heart embryo (6 DAF) expressing the Prom-*Actin*::*GFP*::*ATG8f* revealed the presence of autophagosomes in WT (a) but not in *atg5* (b). Cross-sections of the embryos were incubated with GFP monoclonal antibodies and then with Alexa Fluor 488 secondary antibody (a,b) and with Alexa Fluor 488 secondary antibody only (c,d; single image and e, f; stacked image) as a negative control. Fluorescent signals of the labelled GFP::ATG8f (shown as green dots) were observed by confocal microscopy on 8 plants. Tissue autofluorescence is in red.

Autophagy controls resource allocations and protein storage accumulation in Arabidopsis seeds

Julien Di Berardino, Anne Marmagne, Adeline Berger, Kohki Yoshimoto, Gwendal Cueff, Fabien Chardon, Céline Masclaux-Daubresse and Michèle Reisdorf-Cren

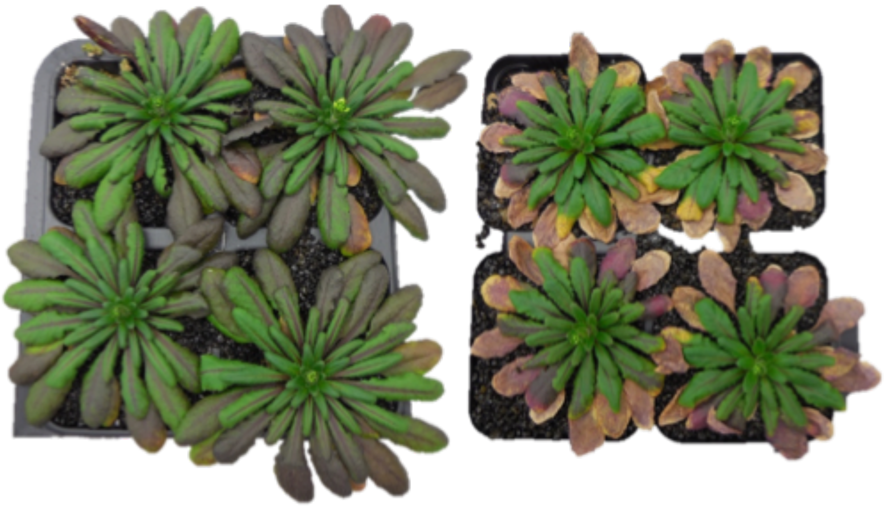

Supplementary Fig. S3. Development of Col and *atg5* rosettes. Pictures represent plants grown in 2mM nitrate conditions, one week after transfer in long day.

Autophagy controls resource allocations and protein storage accumulation in Arabidopsis seeds

Julien Di Berardino, Anne Marmagne, Adeline Berger, Kohki Yoshimoto, Gwendal Cueff, Fabien Chardon, Céline Masclaux-Daubresse and Michèle Reisdorf-Cren

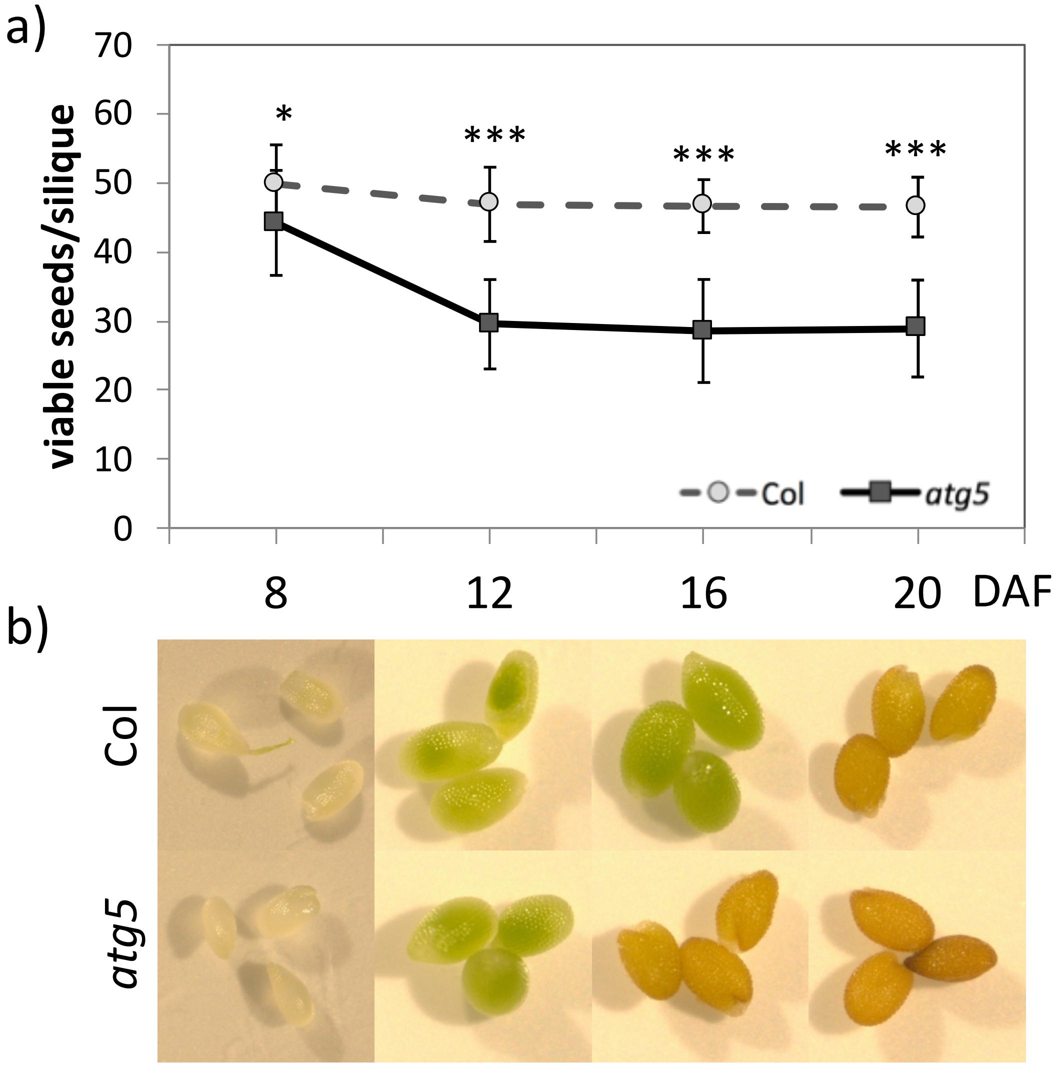

Supplementary Fig. S4. Browning phenotype of *atg5* seeds of plants grown under low nitrate conditions. (a) The number of fully developed viable seeds per silique was monitored during development in wild type (Col) and the *atg5* mutant. Means (± SD) of 20 siliques are shown. *: p-value<5%, ***: p-value<1‰. DAF: Days After Fertilization. (b) Imaging of *atg5* and WT seeds using the same magnification throughout.

Autophagy controls resource allocations and protein storage accumulation in Arabidopsis seeds

Julien Di Berardino, Anne Marmagne, Adeline Berger, Kohki Yoshimoto, Gwendal Cueff, Fabien Chardon, Céline Masclaux-Daubresse and Michèle Reisdorf-Cren

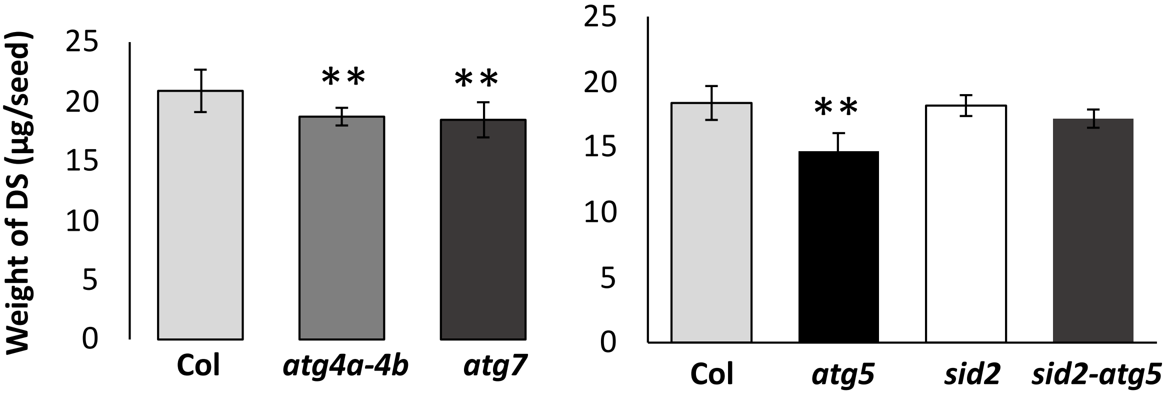

Supplementary Fig. S5. Weight of dry seeds. Seeds of Col, and *atg4a-4b, atg7, atg5, sid2* and *sid2-atg5* mutants, were obtained from the same culture of plants grown under low nitrate conditions. Means (± SD) of three biological replicates are shown. **: p-value<1%. DS: dry seed.

Autophagy controls resource allocations and protein storage accumulation in Arabidopsis seeds

Julien Di Berardino, Anne Marmagne, Adeline Berger, Kohki Yoshimoto, Gwendal Cueff, Fabien Chardon, Céline Masclaux-Daubresse and Michèle Reisdorf-Cren

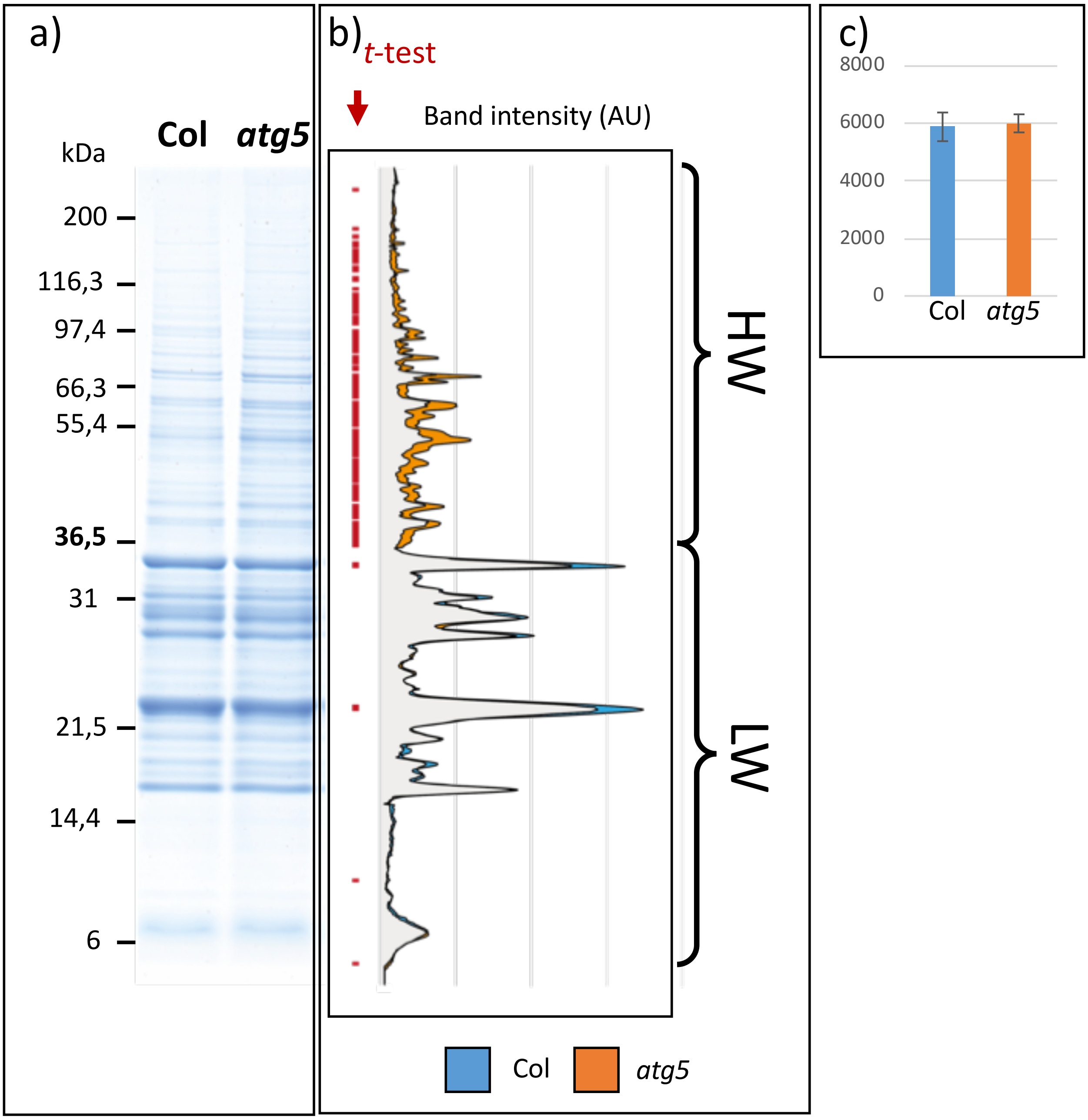

Supplementary Fig. S6. Protein profiles of Col and *atg5* dry seeds produced under low nitrate conditions. Total proteins extracted from dry seeds were separated on SDS-PAGE gels and stained with Coomassie blue (a). The same quantity of protein was loaded in each lane. The graphs in (b) represent the average of the band intensities (AU, arbitrary unit) measured on three biological repeats. Significant differences between Col and *atg5* are indicated by red dots on the left of the (*t*-test, $P < 5\%$). Differences between Col and *atg5* protein contents are presented in orange when the content was higher in *atg5* than in Col, and in blue when the content was higher in Col than in *atg5*. The orange and blue colours correspond to the high-weight (HW, > 37 kDa) and low-weight (LW, < 37 kDa) proteins, respectively. The sum of the band intensities in the Col and *atg5* protein extracts is indicated in (c) as the loading control.

Autophagy controls resource allocations and protein storage accumulation in Arabidopsis seeds

Julien Di Berardino, Anne Marmagne, Adeline Berger, Kohki Yoshimoto, Gwendal Cueff, Fabien Chardon, Céline Masclaux-Daubresse and Michèle Reisdorf-Cren

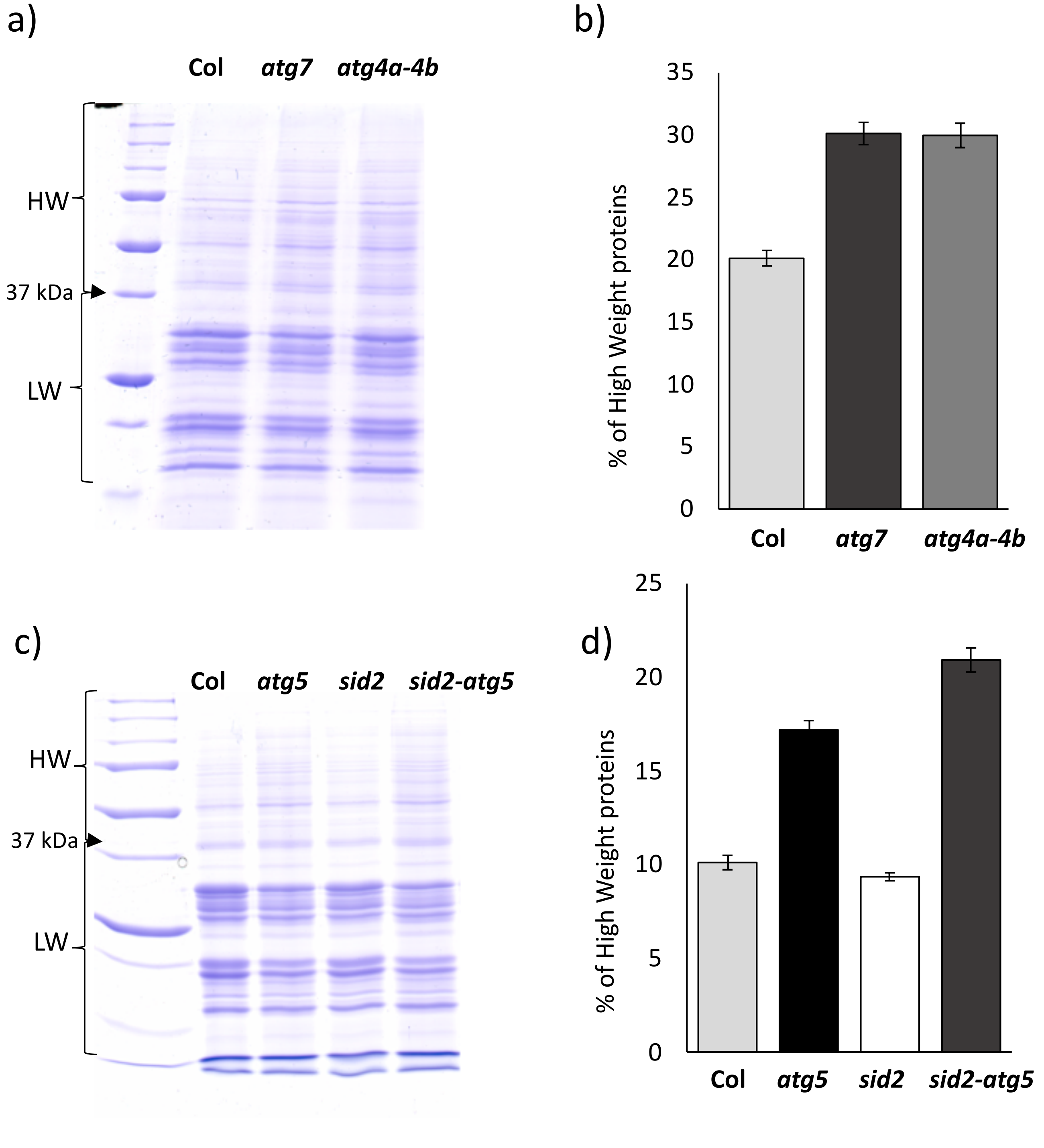

Supplementary Fig. S7. Protein profiles in dry seeds of Col and *atg* mutants. Seeds were produced under low nitrate conditions. SDS-Page gel stained with Commassie blue is presented (a, wild type Col, and *atg7* and *atg4a-4b* mutants; c, wild type Col, and *atg5, sid2* and *sid2-atg5* mutants). Protein loading in each lane was performed on the basis of equal seed quantity. Three biological repeats were analysed. The graphs in (b,d) present the percentage of high molecular weight proteins (HW > 37 kDa) extracted from Col and *atg* dry seeds. LW: low molecular weight proteins (< 37 kDa).

Autophagy controls resource allocations and protein storage accumulation in Arabidopsis seeds

Julien Di Berardino, Anne Marmagne, Adeline Berger, Kohki Yoshimoto, Gwendal Cueff, Fabien Chardon, Céline Masclaux-Daubresse and Michèle Reisdorf-Cren

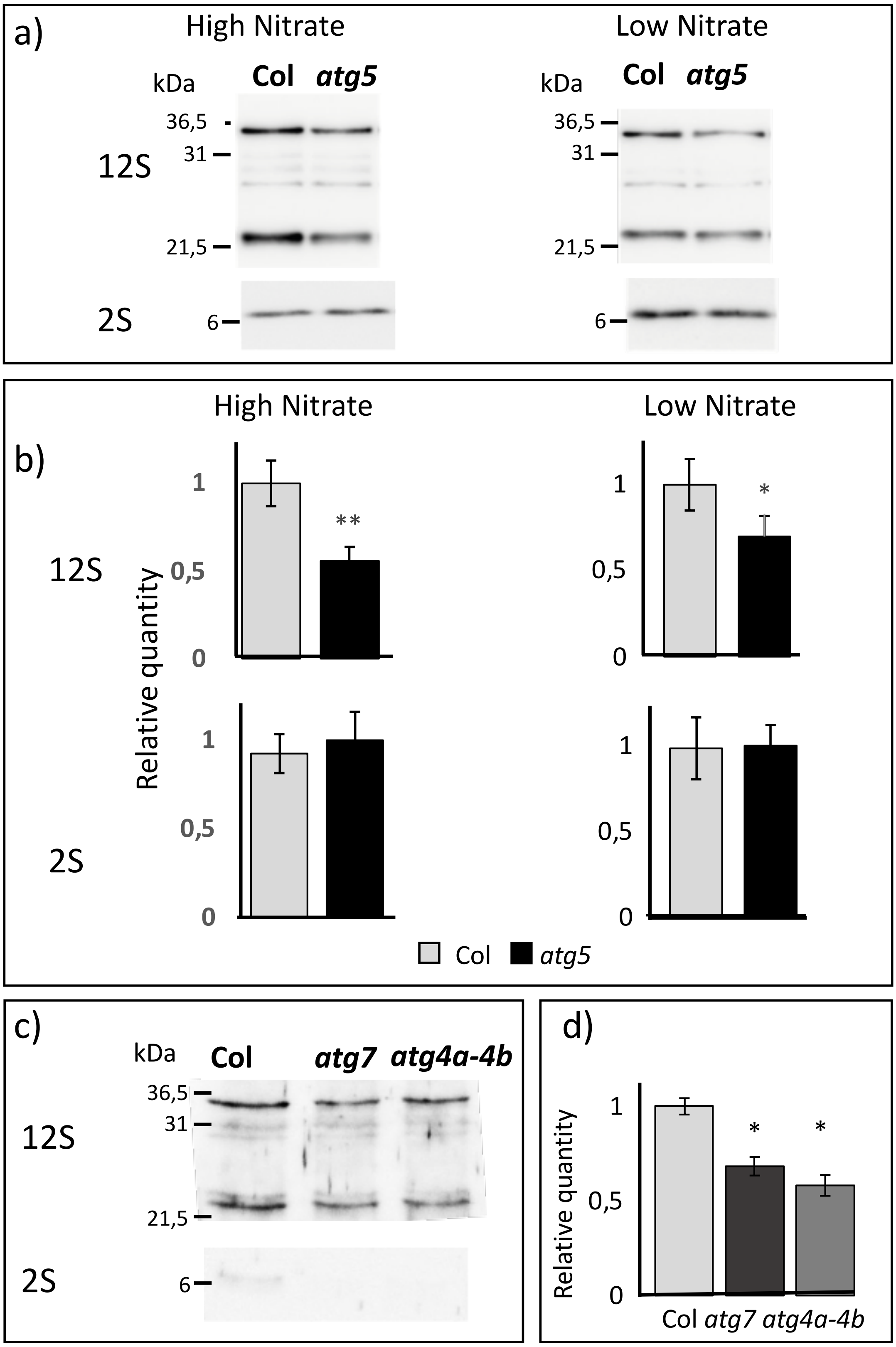

Supplementary Fig. S8. 12S globulin and 2S albumin contents in dry seeds. Seeds were obtained from wild-type Col, *atg*5, *atg7* and *atg4a-4b* mutants grown under high and/or low nitrate conditions. (a, c) Western blots were performed using 12S and 2S storage protein antibodies. (b, d) The means (± SD; *n*=3) of the band intensities quantified with ImageJ software the relative quantity of 12S and 2S storage proteins in dry seed of (b) Col and the *atg*5 mutant under high and low nitrate conditions and (d) Col, *atg7* and *atg4a-4b* mutants under low nitrate conditions. Protein loading in each lane was performed on the basis of the same number of dry seeds. *P<5%, **P< 1%.

Autophagy controls embryo development and protein storage accumulation in Arabidopsis seeds

Julien Di Berardino, Anne Marmagne, Adeline Berger, Kohki Yoshimoto, Gwendal Cueff, Fabien Chardon, Céline Masclaux-Daubresse and Michèle Reisdorf-Cren

Supplementary Table S1: Primers used for RT-qPCR analyses and cloning.

Gene/AGI	Lenght of PCR fragment	Primers 5'→3'	Gene/AGI	Lenght of PCR fragment	Primers 5'→3'
ATG1a/At2g37840	100 pb	F: GTCGATACGAGAATTGAGAGGAAG	ATG10/At3g07525	178 pb	F: TGGAGAACCTCTTGCTTTGG
		R: CGTTTTCCACTTTTTGGTTGAAA			R: ACTTTGGGAAAGCAGCTTGA
ATG1b/At3g53930	108 pb	F: CATCAGCTCAATTTCGGATCT	ATG12a/At1g54210	167 pb	F: TGCTTGCTTTGTTGGAAAGA
		R: GATAACCCTTCCGCTTCTCC			R: CTTTTGTTTAGCCGACGGA
ATG1c/At3g61960	233 pb	F: ACGCTCGTCGTTTTAGCTGT	ATG12b/At3g13970	162pb	F: TTCTAAGACGGCAGCTTCAC
		R: TGAGTGGCAGCACTTGTTTC			R:CGAAGTTTTAACCCCATGCC
ATG2/At3g19190	155 pb	F: TGCAAAATCTGCGTCTATCG	ATG13a/At3g49590	159 pb	F: GAGAAGGTTCAGTGTCAGGT
		R: GTCGAGGACCAGCTTCAGAC			R: TTGATTTGGCTCAGTGGACG
ATG3 /At5g61500	195 pb	F: TCATCCACACTTGCCTGGTA	ATG13b/At3g18770	224 pb	F: TCTCCGCGGATATCATTTTC
		R: CCGAGATCAAAGTCCATTGTG			R: ATTCTCACAAGGGCACCAAC
ATG4b/At3g59950	172 pb	F: CTTTCACGTTCCCTCAAAGC	ATG18a/At3g62770	223 pb	F: AGGAACGGTCCATGTCTTTG
		R: TTGCAATGGTAAGACGATGTG			R: AACGGTGTTCTTTTGATGGC
ATG5/At5g17290	152 pb	F: TAATCGCCCTGTTGAGTTCC	ATG18b/At4g30510	137 pb	F: CTGCTATGAACGAGCTGCTG
		R: TCGACCCATCTGCTTCTTCT			R: AACCCGTTGTGGTCTTGAAC
ATG6/At3g61710	203 pb	F: ATGGGTCTGCCAAAACTGTC	ATG18c/At2g40810	130 pb	F: CTGTTTCAAATCCCCAAGGA
		R: GAGGAATGCCCTGAGATTGA			R: CCAGCTGCAAAACAACTTGA
ATG7/At5g45900	185 pb	F: TCGTGGTTTCGCTGACTTGA	ATG18d/At3g56440	129 pb	F: TGCAGTATCAAGCGACAAGG
		R: CGACAGCAGAAACAGCCAAC			R: CTGGCTGGAGAAACAAGAGG
ATG8a/At4g21980	189 pb	F: CAATTTGTATACGTGGTTCGT	ATG18e/At5g05150	104 pb	F: TGTGGTGGTCCTGAAACAAA
		R: AGCAACGGTAAGAGATCCAA			R: TGACGCAACACAGTCCTTTC
ATG8b/At4g04620	107 pb	F: AACTGCGGCATTGATGTCTG	ATG18f/At5g54730	212 pb	F: TGGAAGGGAGATGGAGATTG
		R: AGCAGTAGAAAGATCCACCAAA			R: CGCTATGTACAGCACCGAGA
ATG8c/At1g62040	110 pb	F: ACTGCTGCAATGATGTCTGC	ATG18g/At1g03380	103 pb	F: CGAGGTGGTCCAGTTTCATT
		R: TGGAAGGCACACATTTAAACC			R: CAGCAACAACCAGCAAAAGA
ATG8d/At2g05630	173 pb	F: ATGTTGTACGGAAGCGGATC	ATG18h/At1g54710	166 pb	F: CAATGGTATGGTTTACCCCG
		R: GAAGAAGATCCCGAACGTGT			R: GACATGAGCGTCGTCGTCTA
ATG8e/At2g45170	101 pb	F: TCTTTAAGATGGACAACGATTTC	VPS15/At4g29380	181 pb	F: TAGCATGGACAAACGAGCAG
		R: CTCAGCCTTTTCCACAATCA			R: GGTACCGACATGGAATGACC
ATG8f/At4g16520	124 pb	F: GCGCTCATGTCTTCTGTGTA	VPS34/At1g60490	170 pb	F: CAGAGCACCGAAGCATAACA
		R: TGAAATATCCAGCAAGAGGTCTC			R: AAGGAGGAGGTTGTCCAGGT
ATG8g/At3g60640	167 pb	F: GGAGCGATGATGTCAACCA	EF-1a/At5g60390	72 pb	F: TGAGCACGCTCTTCTTGCTTTCA
		R: AGCAAGTTCACGGACAAGAA			R: GGTGGTGGCATCCATCTTGTTACA
ATG8h/At3g06420	153 pb	F: AACACTCTTCCTCAAACCGC	APC2/At2g04660	96 pb	F: TTCTGGAAGCAGTGGGTGAA
		R: ATTCAGAGTGCGCTTCGATT			R: CTCCACTTCCATCTGTAAGC
ATG8i/At3g15580	113 pb	F: TGTCAACAACACTCTCCCTCA	ATG8f Promoter	1827 kb	F: ATGCGGTAAATCTGGAGCTA
		R: AACCAAAGGTTTTCTCACTGC			R: CGAAGCTTTTCTGCGATCA
ATG9/At2g31260	240 pb	F: TTGGTGGTCCAAAACACTCA			
		R: GCAAACATGGCCTACACCTT			
